# A Multi-Scale Approach to Microencapsulation by Interfacial Polymerization

**DOI:** 10.3390/polym13040644

**Published:** 2021-02-22

**Authors:** Fabián Ricardo, Diego Pradilla, Ricardo Luiz, Oscar Alberto Alvarez Solano

**Affiliations:** 1 Grupo de Diseño de Productos y Procesos (GDPP), Department of Chemical and Food Engineering, Universidad de los Andes, Bogotá 111711, Colombia; fd.ricardo@uniandes.edu.co (F.R.); d-pradil@uniandes.edu.co (D.P.); 2 The Dow Chemical Company, Dow Brasil Sudeste Industrial Ltda, Sao Paulo 04794-000, Brazil; RVLuiz@dow.com

**Keywords:** microencapsulation, interfacial polymerization, multi-scale analysis, controlled release, compressive strength, aggregation

## Abstract

This work applies a multi-scale approach to the microencapsulation by interfacial polymerization. Such microencapsulation is used to produce fertilizers, pesticides and drugs. In this study, variations at three different scales (molecular, microscopic and macroscopic) of product design (i.e., product variables, process variables and properties) are considered simultaneously. We quantify the effect of the formulation, composition and pH change on the microcapsules’ properties. Additionally, the method of measuring the strength of the microcapsules by crushing a sample of microcapsules’ suspension was tested. Results show that the xylene release rate in the microcapsules decreases when the amine functionality is greater due to a stronger crosslinking. Such degree of crosslinking increases the compression force over the microcapsules and improves their appearance. When high levels of amine concentration are used, the initial pH values in the reaction are also high which leads to agglomeration. This study provides a possible explanation to the aggregation based on the kinetic and thermodynamic controls in reactions and shows that the pH measurements account for the polyurea reaction and carbamate formation, which is a reason why this is not a suitable method to study kinetics of polymerization. Finally, the method used to measure the compressive strength of the microcapsules detected differences in formulations and composition with low sensibility.

## 1. Introduction

Microcapsules consist of particles which entrap an active ingredient within a polymeric or proteinic structure [1]. In matrix-type microspheres, the structure is present throughout the entire volume of the microcapsule, while in core-shell particles, the envelope forms a shell around a nucleus. Microencapsulation provides different functionalities, for instance, they (i) protect the encapsulated active ingredient from external factors; (ii) allow a controlled release of the substance of interest; (iii) prevent waste or degradation of the substance to improve the cost-benefit ratio of the product; and (iv) reduce impacts to the environment and human health by targeting a specific place in the system of interest [1,2,3]. There is a wide variety of methods to encapsulate a substance which includes spray drying, pan coating, lyophilization, ionic gelation, solvent evaporation, coacervation, interfacial polymerization, vibrational nozzle and centrifugal extrusion [4,5,6]. Interfacial polymerization is a microencapsulation method widely used for the production of pesticides [7,8], phase change materials [9,10], cosmetics and pharmaceutics [11] mainly due to the high active ingredient (AI) loading that can be achieved (typically 480 g of AI/liter in the case of pesticides) [8], a synthesis that is moderately fast, high yield, controllable release characteristics and easy processing steps [12]. 

In order to understand the phenomena involved in microencapsulation by interfacial polymerization, a multi-scale approach for its study is put forward in this work. Through this approach, a coupled analysis of three different scales (i.e., molecular, microscopic and macroscopic) for product design is established. First, product variables, that is, variables that represent changes in the type of substances added (formulation) and the amount used (composition); second, process conditions (e.g., temperature, pressure, time of reaction, pH), and third, the final properties of the product. This analysis makes it possible to link the phenomena involved in a system with its performance as a finished product, providing a holistic perspective in product design [13]. 

The literature is rich in the use of multi-scale approaches that allowed explaining phenomena and proposing relationships between process conditions, product variables and product properties [13,14,15,16,17,18,19]. Relationships between different scales have been proposed for microencapsulation by interfacial polymerization [20,21]. To cite some examples, Johnsen and Schmid [22] prepared microcapsules with a polyurethane wall using interfacial polymerization. They found that the formulation (i.e., nature of the encapsulated substances, the surfactants and the isocyanate used) and the process conditions (i.e., pH, salinity, temperature and type of catalyst), had an impact on the macroscopic properties (i.e., the size of the microcapsules and the agglomeration dynamics). S.J. Wagh et al. [23] carried out a study in which correlations were found among product variables (i.e., phase volume ratio, amine and isocyanate content and the type of solvent), a process variable (i.e., pH values during the reaction) and other properties (i.e., molecular weight of the polyurea formed over the capsules and crystallinity of the polymers). The analysis allowed establishing that the rate of polymerization is inversely proportional to the crystallinity of the polymer formed; the reaction rate depends on the polarity of the solvent, the molecular weight of the polymer shows a dependence on the molar ratio of reactants and the monomer consumption rate shows a first order dependence on the amine concentration. Siddhan et al. [24] encapsulated n-octadecane mixed with cyclohexane and used toluene-2,4-diisocyanate (TDI) and diethylene triamine (DETA) as reagents. They concluded that the composition (i.e., core/amine ratio and n-octadecane/cyclohexane ratio) and the process conditions (i.e., mixing speed in each stage, temperature and use of catalyzer) had influence on the properties (i.e., efficiency of the encapsulation and the stability of the microcapsules when heating and washing as well as the agglomeration) of the microcapsules for the application as phase change materials. It is clear that the main advantage in performing studies over different scales may lead to find and understand phenomena that otherwise would have remained obscure if the traditional approach of one variable and one effect would have been followed. 

The goal of this work is to use a multi-scale analysis on the encapsulation of xylene by interfacial polymerization to study the possible relationships between two product variables (i.e., formulation and composition), one process variable (pH variation) and four final properties at two different levels, namely, wall imperfections and agglomeration of the microcapsules (microscopic) and xylene release rate and compressive strength (macroscopic). The xylene was selected as the core material due to its importance in encapsulation of pesticides and bactericides [25,26]. 

Within the framework of this multi-scale approach analysis, the validity of the pH measurement method reported in previous studies as suitable to follow reaction kinetics or diffusion of reagents over the wall of the microcapsules is tested [23,27,28]. Furthermore, while some studies measure the strength of the polymer forming the shell by synthetizing the polymer separately and using tensiometry or two-dimensional rheology [29,30], or directly on the microcapsule by using micromanipulation [31] and nanomanipulation [32]; here, the measurement is carried out over a sample of the microcapsules’ suspension with a straightforward procedure using a texture analyzer. To the best of the authors´ knowledge, this is the first time that this technique is used to follow the compressive force directly on the suspension of microcapsules instead of isolating them and testing their strength separately. This simple method may not be suitable to quantify the effect of the applied forces on deformation, breakage or release. However, its usefulness for studying the general trend in the strength of the walls of the microcapsules as a function of formulation changes is evaluated. The basis for these remarks is that in terms of final product performance, the average interaction of the microcapsules is the determining parameter. Finally, despite there being studies that report agglomeration of microcapsules [24,33,34,35], no mechanistic explanations of this phenomenon are reported. A hypothesis based on the kinetic and thermodynamic control of chemical reactions is formulated to understand agglomeration dynamics of the microcapsules as a function of the variations in the formulation and composition. 

Encapsulations of xylene were performed using an isocyanate and two amines with different functionalities in order to analyze the effect of the type of amine on the properties. Three different concentrations of both reagents, amine and isocyanate, were tested while maintaining their ratio to study the influence of the product’s composition. The pH values at the beginning and the end of the reaction were monitored to quantify the effect of this variable on the other scales.

## 2. Materials and Methods 

### 2.1. Materials

**Figure 1:**
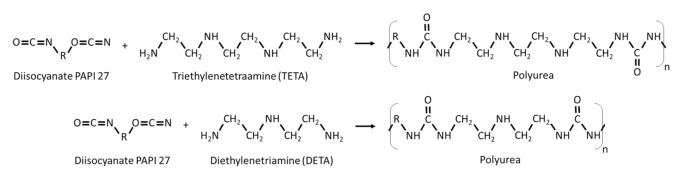
Chemical reactions of DETA and TETA with the diisocyanate PAPI 27 to form polyureas with different molecular structure.

Polyethylene glycol sorbitan monolaurate 20 (Tween 20) and Sorbitan monooleate 80 (Span 80) (provided by Croda, Snaith, UK) were used as emulsifiers. HPLC grade o-xylene (PanReac AppliChem, Chicago, IL, USA) was used as the oil phase. The isocyanate for the reaction was PAPI^TM^ 27 (Dow Chemical Co., Midland, MI, USA). This is a polymethylene polyphenylisocyanate with 2.7 of functionality. The amines used to crosslink the isocyanate were diethylene triamine (DETA) with a functionality of 3 and triethylene tetramine (TETA) with a functionality of 4 (provided by Dow Chemical Co., Midland, MI, USA). Figure 1 shows the reaction between DETA, TETA and PAPI 27. It is worth noting that PAPI 27 is a mixture of diisocyanates, which means that the “R” group of the PAPI 27 showed in Figure 1 contains amino groups for some molecules so that the functionality can be averaged 2.7. Acetone which was used for the extraction of the encapsulated xylene, came from PanReac AppliChem, Chicago, IL, USA.

### 2.2. Formulation

The amount of encapsulated xylene was 12 g while the amount of water (continuous phase) was 186 g. An HLB test was developed to rapidly locate the point where stability reaches a maximum. Stability is defined here as the timeframe where no visual phase separation is observed within the experimental window. By preparing emulsions of varying Tween 20/Span 80 proportion and keeping all the other formulation and composition variables constant, it is possible to identify the adequate surfactant ratio that allowed more stability (i.e., no evident phase separation) during the testing phase. The HLB value required by the emulsions in this case was 11, which means that the necessary Tween 20/Span 80 ratio by weight was 1.172 with a total mass of the surfactants at 0.96 g.

The amounts of isocyanate and amine added to each formulation with its respective abbreviation can be seen in Table 1.

### 2.3. Manufacturing of Microcapsules

First, the phases of the emulsion were mixed separately. The aqueous phase, consisting of water and Tween 20, was mixed using a homogenizer (Dispermat CV-2 VMA-GETZMANN GMBH, Reichshof, Germany) with a dispersing and homogenizing blade at 1000 rpm in a 400 mL tank. The organic phase consisted of xylene, Span 80 and the required amount of PAPI^TM^ 27, and it was homogenized in a 100 mL tank using a magnetic stirrer at 660 rpm. The homogenization process for each phase took 5 min. Later, the velocity of the Dispermat homogenizer was increased to 2500 rpm and the organic phase was added manually to the aqueous phase. The emulsification time was 5 min.

**Figure 2:**
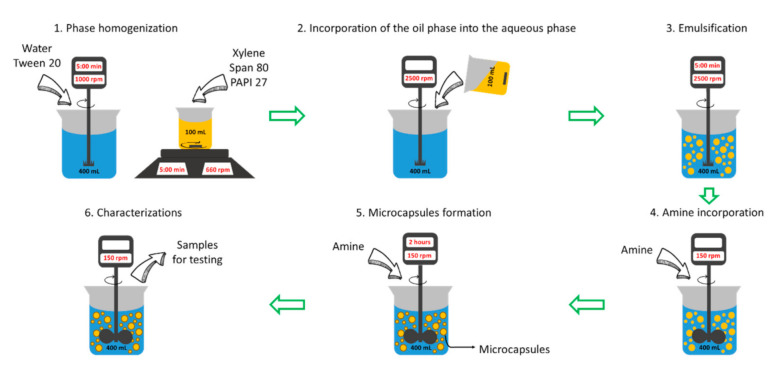
Schematic representation of the preparation process of the microcapsules. The temperature of the environment was 18 °C.

Once the emulsion was obtained, it was transferred to a mixing device (IKA^®^ Eurostar Power, Staufen, Germany) with a propeller where crosslinking took place. The established amount of amine solution was added to the emulsion and allowed to crosslink for 2 h at 150 rpm. The temperature of this manufacturing process was 18 °C. After that, characterization was carried out for each formulation. A detailed schematic representation of this process is shown in Figure 2. All systems were prepared and characterized by duplicate.

### 2.4. Initial and Final pH Value

Initial pH was registered 10 min after amine addition and the final pH was registered after 2 h of crosslinking for all systems. This was done with the suspension of microcapsules and with a reference sample (blank), which consisted of the same formulation as in Table 1 without diisocyanate. The blank was used to guarantee that the pH values corresponded to the reaction process and not to other sources. The pH values were taken using a pH meter (Mettler Toledo SG23-SevenGo Duo, Columbus, OH, USA).

### 2.5. Optical Microscopy

Images that allowed to visually evaluate the shape of the microcapsules, as well as aggregation dynamics were taken with an optical microscope (Motic^®^ BA310, Xiamen, China) at 40×. The amount of sample added for the images was approximately 0.01 mL.

### 2.6. Xylene Release Rate of the Microcapsules

Due to the high volatility of xylene, the release rate was evaluated by measuring the xylene vaporization under certain conditions. Samples of the microcapsule suspension were introduced in 6 watch glasses and put inside a climate chamber RGX-250E (Huanghua Faithful Instrument Co., Huanghua, China) at 25 °C and a relative humidity (RH) of 80%. Over the course of 3 h, the samples were taken out in 30-min intervals. Their contents were immediately transferred to test tubes in which acetone was added to extract the xylene inside the microcapsules, as indicated by Tsuda et al. [20]. Each tube with their respective sample and acetone was sonicated for 10 min. Then a small amount of sample was filtered, analyzed and final concentration determined using a gas chromatography unit with a column TRB-1 (Shimadzu GC-2010, Kyoto, Japan).

### 2.7. Compressive Strength of the Microcapsules

Compression tests were carried out using a texture analyzer TA.HD plus (Stable Microsystems, Godalming, UK) with a cylindrical probe P/35. The procedure was applied to a population of microcapsules. A well-mixed suspension sample of 0.3 mL was taken and placed in the center of the texture analyzer. Then, a compression procedure was set to lower the probe at a velocity of 120 mm/min, until the final gap value of 1 µm between the probe and the base (gap of maximum compression) was achieved. The instrument reports data of force vs. distance from the base. The maximum value of the force, which occurs at the final gap distance, is taken in order to compare with the other formulations.

## 3. Results and Discussion

Within the framework of the multi-scale approach proposed here, an analysis is carried out taking into account all the characterizations described above and yielding correlations between the variables of the product (i.e., type of amine, formulation and amount of reagents, composition), process (i.e., pH change during reaction) and properties (i.e., xylene release rate, form, agglomeration and compressive strength). These correlations are in agreement with previously reported results.

### 3.1. Initial and Final pH

**Figure 3:**
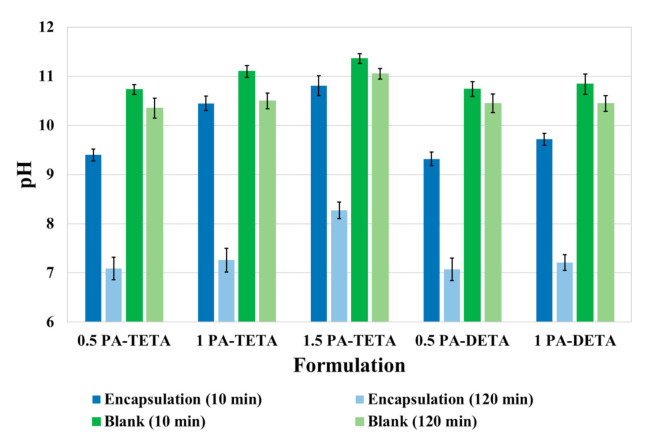
pH values for the different formulations (blue color), 10 min and 120 min after the amine was added to the system. Additionally, the “blanks” for each formulation are also showed (green color) in the same time interval.

The pH values after 10 and 120 min of crosslinking for each formulation and their respective blank are shown in Figure 3. The x-axis contains the name of the formulations tested and the y-axis represents the pH values measured. These values vary approximately from 7 in encapsulations where almost all the amine in the aqueous phase was consumed, to 11 at the beginning of the test in the blanks where more amine was used.

From Figure 3, it can be noticed that the pH values of the blanks decrease with time even though a crosslinking reaction is not supposed to be occurring since no isocyanate was added. The explanation for this lies in the fact that primary and secondary amines react with carbon dioxide present in the air, producing carbamates which make the solution less alkaline and therefore decrease the pH value [36,37]. In other words, the pH reduction of the microcapsules’ suspensions over time reflects two phenomena, amine decreasing due to the crosslinking with the isocyanate and the reaction of the amine with carbon dioxide present in the air. That happens for the encapsulations with DETA as well as with TETA [38]. This fact has been ignored in prior studies in which pH measurements were used to analyze reaction kinetics of amines and isocyanates without taking into account the secondary reaction to form carbamates leading to noisy results [23,27,28]. For example, in the study of Kubo et al. [27], conversions of the amines are taken directly from the pH data and even a diffusion model is proposed without considering the secondary reaction with the CO_2_ of the environment reported here. Even though the total change in pH for the blanks seems low (e.g., from 11.36 to 11.05 for the formulation 1.5 PA-TETA), it is important to note that a pH decrease in the high pH region represents a significant change in the [H+] concentration compared to the neutral pH region (7-8). This strongly suggests that the concentration of amine had a significant variation, due to the reaction with CO_2_ despite the small change in pH. 

It is also noticeable that the pH values at 10 min of reaction are greater as long as more amine is added to the system, which leads to the observation that the more reagents are included to the formulations, the higher pH values are present in the formulation during the reaction. It is worth noting that almost all formulations tend to have a final pH value (120 min) of approximately 7, except for the formulation with the highest amount of reagents (1.5 PA-TETA) in which the final pH was above 8. This high value can be due to the fact that the thickness of the capsule wall is so high, that the diffusion of the reagents through the membrane is sufficiently slow to lower the pH.

### 3.2. Optical Microscopy

**Figure 4:**
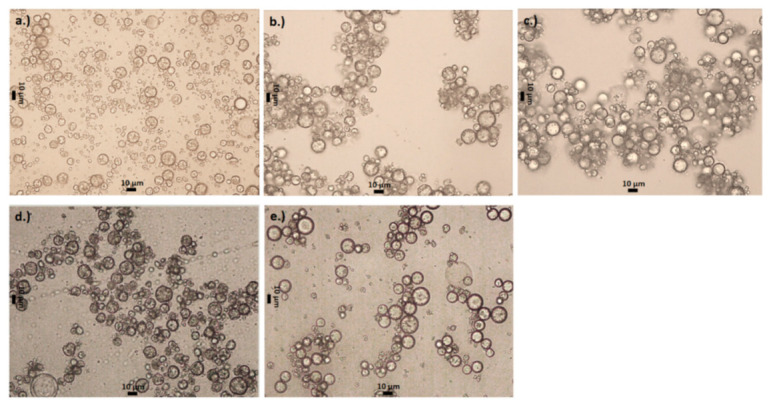
Microscopy images of capsules in which the difference of appearance on the microcapsules’ wall as a function of the formulation can be noted for: (**a**) 0.5 PA-TETA; (**b**) 1 PA-TETA; (**c**) 1.5 PA-TETA; (**d**) 0.5 PA- DETA and (**e**) 1 PA-DETA.

Figure 4 shows images of the microcapsules formed using the two amines, DETA and TETA at the different concentrations of amine and isocyanate, and the morphology of the microcapsules as well as their aggregation can be evaluated for each formulation.

When the lowest concentrations of reagents are used (0.5 PA-TETA and 0.5 PA-DETA), the microcapsules produced show imperfections in their walls (i.e., a smooth interface is not observed), imperfections in their shape (i.e., not completely spherical), some exhibit holes and others exhibit a golf ball-like surface (Figure 4). Microcapsules with these imperfections have been found in other studies where xylene and other volatile components were encapsulated [39,40,41]. The explanation for this shrinkage of the microcapsules is that when the encapsulated substance is volatile, its vaporization creates void spaces for which the capsule wall compensates by shrinking and taking on a more flattened shape [40]. This confirms that the wall of the microcapsules formed by interfacial polymerization could be treated as semipermeable.

Figure 4 also shows that in the formulations 1 PA-TETA and 1 PA-DETA, the microcapsules present fewer imperfections compared to the formulations 0.5 PA-TETA and 0.5 PA-DETA. Similarly, the 1.5 PA-TETA formulation produced completely spherical microcapsules with fewer imperfections. From these differences in the appearance of the microcapsules at the microscopic level, it can be said that defects in shape occur when the amount of polymer at the wall is low, which is a result of a low amount of the reagents [40]. This is because less polyurea forming the walls of the microcapsules with lower reagent concentrations leads to less firmness. Thus, the walls are more easily deformed in the presence of an external stimulus such as xylene liberation or the simple agitation of the suspension of microcapsules during the crosslinking stage. In fact, the phenomenon of shrinkage at the wall of the microcapsules has been related to flexible structures [42]. 

Siddhan et al. [24] showed that agglomeration is reduced when a high stirring velocity (2500 rpm) is applied to the microcapsules during the first 5 min of polymerization and a low velocity is applied in the following 45 min. Taking this into account, the low stirring velocity used here (150 rpm) could be responsible for increasing the agglomeration. However, even when the same stirring velocity was used for all the experiments, in Figure 4, the higher tendency of the microcapsules to agglomerate when the amount of reagents is higher for both amines can be observed. The formulations in which there is no agglomeration are those with the lowest quantity of reagents: 0.5 PA-TETA and 0.5 PA-DETA. It can also be seen that the tendency to agglomerate is higher in the formulations with TETA than with DETA. The reason for the greater agglomeration with TETA is that it has one extra secondary amino group compared to DETA, so the microcapsules that are synthesized with TETA are more likely to crosslink with the surfaces of the neighboring capsules because they have more unpaired secondary groups [43], hence, making them more likely to aggregate or merge.

The tendency of the microcapsules to agglomerate increases with the amount of reagents (Figure 4) since a larger concentration of reactants favors competitive reactions due to a more disordered system. It may be explained in the following way: In chemical reactions, reagents may take different reaction paths and form different products through competitive reaction mechanisms. There are two factors which affect the formation of one product over the other: the thermodynamic and kinetic control [44]. The thermodynamic control basically favors the formation of the product more stable, energetically, while the kinetic control allows the formation of the other products as a consequence of a more disordered reaction caused by the increase of concentration, temperature, etc. In this case, the thermodynamic control favors the reaction of the isocyanate with the primary amine groups located at the ends of the amine molecule because they are more reactive than the secondary amine groups inside the molecular chain. However, when the concentration of the amines increases, the tendency of the isocyanate to react with the secondary amine groups grows accordingly, due to the disorder in the system. For this reason, higher concentration of reagents increases the selectivity of the isocyanate to crosslink with the secondary amino groups in a higher frequency compared to the case of primary amines. By increasing the reaction between the isocyanate molecules and the secondary amino groups, both for DETA and TETA, the primary groups are free and can react in a stronger way with the surfaces of the neighboring microcapsules, increasing agglomeration. This aggregation was previously observed in micro-encapsulations [24,33,34,35], however, these studies focused on preventing or reversing this phenomenon instead of attempting a mechanistic understanding.

### 3.3. Xylene Release Rate of the Microcapsules

**Figure 5:**
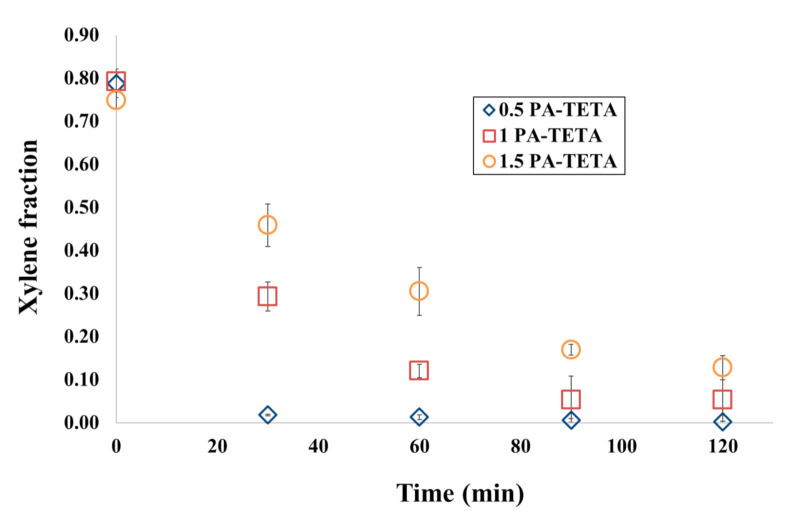
Differences in the kinetics of xylene release at 80% RH and 25 °C for changes in composition of the formulations with TETA and PAPI^TM^ 27.

**Figure 6:**
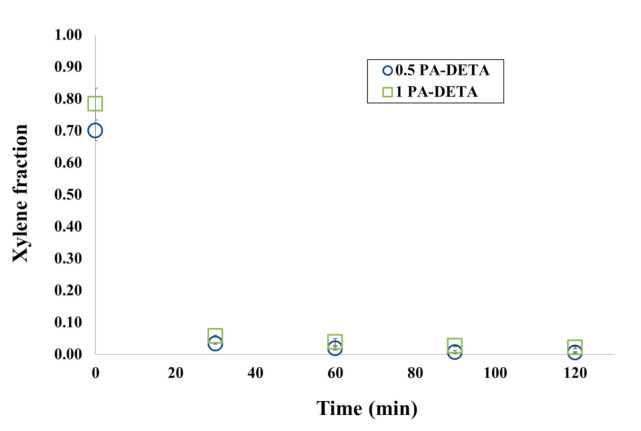
Differences in the kinetics of xylene release at 80% RH and 25 °C for changes in composition of the formulations with DETA and PAPI^TM^ 27.

The xylene fraction as a function of time for the two different amines used in this work (kinetic release of xylene in the tests with TETA and DETA) can be seen in Figure 5 and Figure 6. In these figures, the xylene fraction refers to the amount of xylene obtained in the tests (chromatography) divided into a theoretical amount. This amount corresponds to the fraction that should be obtained if there is no release according to a simple mass balance. As expected, the fraction of xylene lowered during time due to the release. Figure 5 shows that the microcapsules created with higher amounts of reagents delay the release of xylene, indicating a greater amount of polyurea produced at the wall. This is due to the fact that with a greater amount of polyurea, there is a greater physical impediment to the xylene crossing the wall of the microcapsules to reach the exterior. In general, permeation through thicker walls takes longer [45].

Figure 6 shows the kinetics of xylene release for the DETA and PAPI^TM^ 27 formulations. A slight delay in the release as the amount of reagent increases can be observed just as it was seen for the formulations with TETA. However, this delay due to the increase in the amount of reagents is lower when the DETA amine was used than when the same quantities of reagents were tested for TETA. This difference is because the amine TETA has a functionality of 4, while the amine DETA has 3. The amine TETA has greater functionality (i.e., more amino groups), so it can reticulate with more isocyanate chains and achieve a higher crosslinked network giving it stronger mechanical properties and lower xylene release rate at the wall. In previous studies, it was found that by using monomers with greater functionalities, more resistant or less fragile polymeric walls are achieved in the microcapsules [43,46].

Another aspect that can be observed in Figure 5 and Figure 6 is that only in formulations with the lowest amount of reagents (0.5 PA-TETA and 0.5 PA-DETA), the remaining fraction of xylene in the microcapsules was approximately zero at 120 min. The other formulations did not reach the xylene fraction value of zero at 120 min when the release stopped; this is as a consequence of the aggregation of the microcapsules, a phenomenon only absent in formulations with the lowest concentration of reagents, as can be seen in Figure 4. In the rest of the formulations, the microcapsules aggregated forming clusters in which the release occurs mainly in the outer microcapsules of the cluster. When those microcapsules are dried, the clusters hardened to the point that prevented the microcapsules within the cluster, even those with xylene, to continue their release. This is why the final values of the concentration of xylene in the formulations that show aggregation are never zero, but are stabilized at a number corresponding to the amount of xylene that could no longer be released due to the hardening of the surface in the clusters.

### 3.4. Compression Force of the Microcapsules

**Figure 7:**
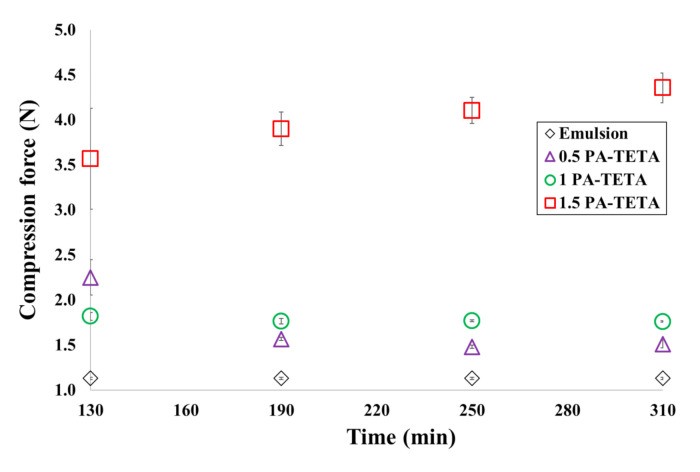
Compression force for the microcapsules of the formulations with TETA and PAPI^TM^ 27 in comparison to the compression force applied for the emulsion, just before the beginning of the polymerization.

**Figure 8:**
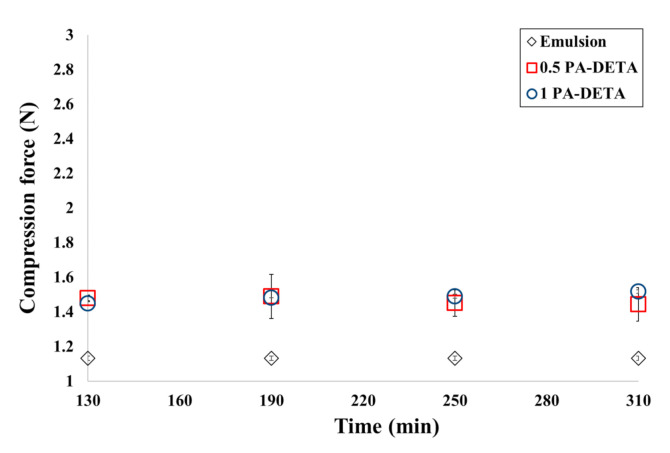
Compression force for the microcapsules of the formulations with DETA and PAPI^TM^ 27 in comparison to the compression force applied for the emulsion, just before the beginning of the polymerization.

The compression force of the microcapsules was measured based on the maximum force required to compress the sample up to the specified gap. This is the force reported by the texture analyzer when the test probe was at the minimum distance from the base (1 μm) with the suspension of microcapsules pressed in the middle. Later, this value was plotted for each formulation as a function of time (measured after 130 min of crosslinking and then every 60 min). These results can be seen in Figure 7 for formulations with TETA and in Figure 8 for formulations with DETA.

The maximum compression force increases as a result of the encapsulation as evidenced in Figure 7. This is because the compression forces of the three formulations are larger than the force required to compress the same amount of sample, but with the emulsion before crosslinking. This is evidence that the difference detected by the instrument in each formulation is a consequence of the formation of the capsule walls around the xylene cores. Figure 7 also shows that the maximum compression force increases as the quantity of reagents increases since there is more polymer at the wall, so the texture analyzer has to compress a larger polyurethane mass, and then a larger force is required to reach the same distance of 1 μm by the probe.

Figure 7 shows that the maximum force values tend to remain constant after two hours of crosslinking, leading to the conclusion that there were no substantial changes in the structure of the wall that could affect the compressive strength of the microcapsules. However, in the formulation with the highest amount of reagent (1.5 PA-TETA), it is possible to see that the compression force continues to slightly increase even after five hours of reaction time. This is due to the fact that after two hours, the reaction continued to proceed slowly because it was difficult for the reagents to diffuse through the wall; therefore, the force needed to compress the wall also continued to progressively increase as the wall became thicker. This observation is consistent with the behavior of xylene release for the formulation 1.5 PA-TETA shown in Figure 5, where it can be noticed that such formulation showed the slowest rate of release compared to the other two formulations. In other words, the slow mass transfer of chemical substances inside the microcapsules through the wall for the 1.5-PA-TETA formulation was reflected in the delay of xylene release and the slower flux of reagents through the wall that produced a change in the compression force over time.

Analyzing Figure 8, when PAPI^TM^ 27 and DETA were used, the compression force also increased due to the formation of polymer at the wall of the capsules since the measurements for the encapsulation have greater compression force than the ones for the emulsion. However, it should be noted that error bars do not allow to see a difference between the compression forces in the formulations 0.5 PA-DETA and 1 PA-DETA. This means that the difference between those formulations cannot be detected within the sensibility of the method used to measure the compression force in the microcapsules.

Finally, in the formulations with TETA, the compression forces are greater than with the DETA amine. As mentioned earlier, this occurs because the TETA amine has a greater functionality which turns into a higher degree of crosslinking with the isocyanate, improving the mechanical properties.

## 4. Conclusions

Different multi-scale studies have been conducted on the field of microencapsulation by interfacial polymerization where relations between product, process and property variables were found [22,23,24]. Analyzing the three scales is important in product and process design, especially because one change on just one scale produces changes on the others simultaneously.

This work reports on a multi-scale approach analysis for the production of xylene microcapsules using the interfacial polymerization method with an isocyanate and two amines. This method of encapsulation has been extensively used for various applications [7,8,9,10,11], and having a broad knowledge about the phenomena involved at the three scales studied that affect its performance is important for integral product design. By making changes in the product variables (i.e., formulation and concentration of the reagents), it was possible to analyze the effects on the pH at the initial and final stages of the reaction (process variable), the compressive strength and xylene release rate (macroscopic properties) and the imperfections and aggregation (microscopic) of the microcapsules’ suspension. 

Through this multi-scale analysis, it was possible to find links between the studied scales. The results found here are consistent with others obtained in past studies, such as the (i) reduction of pH shown in Figure 3 as a consequence of the polymerization, (ii) the agglomeration of microcapsules at low stirring speeds, (iii) the presence of deformations, holes and indentations over the wall of the microcapsules (Figure 4), (iv) the semipermeable nature of the microcapsules produced by interfacial polymerization reflected on the release of xylene (Figure 5 and Figure 6), (v) the slower release of the core ingredient as a consequence of thicker walls, and (vi) the increase of the microcapsules’ strength due to more polymers present (Figure 7 and Figure 8). 

However, data obtained also allowed to find out that several authors used pH values to study the interfacial reaction to form polyurea microcapsules [23,24,27,28], but they did not consider the side reaction of amines with carbon dioxide to form carbamates—something that was detected in this study. The method used here, different to other reported techniques [29,30,31,32], allowed to measure the compression force to a sample of microcapsules and this also permitted to find coherent results for changes in formulation and composition. However, the sensibility of the method may be an obstacle for some applications and only provides a way to compare the strength of different formulations of the polymer in the wall of the microcapsules. Finally, it was possible to provide a hypothesis for the aggregation of the microcapsules based on the thermodynamic and kinetic control of chemical reactions, analyzing the phenomenon along with the molecular structure of the amines used and their amounts.

The explanation provided for the agglomeration must be developed further. This may include setting the pH value in the reaction by means of a buffer solution and using other amines with different functionalities and compositions.

## Figures and Tables

**Table 1:** Abbreviations of the formulations, molar amounts and isocyanate/amine molar ratios.

Formulation	Abbreviation
0.5 g PAPI^TM^ 27 (1.47 mmol PAPI) and 0.5 mL solution TETA 12% *v*/*v* of amine/water (0.40 mmol TETA). Isocyanate/amine ratio: 3.65	0.5 PA-TETA
1 g PAPI^TM^ 27 (2.94 mmol PAPI) and 1 mL solution TETA 12% *v*/*v* of amine/water (0.80 mmol TETA). Isocyanate/amine ratio: 3.65	1 PA-TETA
1.5 g PAPI^TM^ 27 (4.41 mmol PAPI) and 1.5 mL solution TETA 12% *v*/*v* of amine/water (1.21 mmol TETA). Isocyanate/amine ratio: 3.65	1.5 PA-TETA
0.5 g PAPI^TM^ 27 (1.47 mmol PAPI) and 0.5 mL solution DETA 12% *v*/*v* of amine/water (0.56 mmol DETA). Isocyanate/amine ratio: 2.65	0.5 PA-DETA
1 g PAPI^TM^ 27 (2.94 mmol PAPI) and 1 mL solution DETA 12% *v*/*v* of amine/water (1.11 mmol DETA). Isocyanate/amine ratio: 2.65	1 PA-DETA

## Data Availability

All relevant data are within the manuscript.
